# PRIME-HCC: phase Ib study of neoadjuvant ipilimumab and nivolumab prior to liver resection for hepatocellular carcinoma

**DOI:** 10.1186/s12885-021-08033-x

**Published:** 2021-03-23

**Authors:** David J. Pinato, Alessio Cortellini, Ajithkumar Sukumaran, Tom Cole, Madhava Pai, Nagy Habib, Duncan Spalding, Mikael H. Sodergren, Maria Martinez, Tony Dhillon, Paul Tait, Robert Thomas, Caroline Ward, Hemant Kocher, Vincent Yip, Sarah Slater, Rohini Sharma

**Affiliations:** 1 Division of Cancer, Department of Surgery & Cancer, Imperial College London, Hammersmith Campus, Du Cane Road, W120HS, London, UK; 2 Department of Translational Medicine, Università del Piemonte Orientale “A. Avogadro”, Via Paolo Solaroli, 17, 28100 Novara, NO Italy; 3 Department of Biotechnology and Applied Clinical Sciences, University of L’Aquila, Via Vetoio, 67100 L’Aquila, Italy; 4 NIHR Imperial CRF, Imperial College London, Hammersmith Hospital, Du Cane Road, W120HS, London, UK; 5 Division of Surgery, Department of Surgery & Cancer, Imperial College London, Hammersmith Hospital, Du Cane Road, W120HS, London, UK; 6 Faculty of Health and Medical Sciences, University of Surrey and Department of Oncology, The Royal Surrey Hospital, Egerton Rd, Guildford, GU2 7XX UK; 7 Department of Radiology, Imperial College NHS Trust, Hammersmith Hospital, Du Cane Road, W120HS, London, UK; 8 Barts and The London HPB Centre, The Royal London Hospital, Barts Health NHS Trust, London, UK; 9 Centre for Tumour Biology, Barts Cancer Institute, Queen Mary University of London, London, UK; 10 Department of Medical Oncology, Barts Health NHS Trust, London, UK

**Keywords:** HCC, Immunotherapy, PD-1, PD-L1, CTLA-4

## Abstract

**Background:**

After liver resection (LR), patients with hepatocellular cancer (HCC) are at high risk of recurrence. There are no approved anti-cancer therapies known to affect such risk, highlighting the acute need for novel systemic therapies to control the probability of disease relapse. Immunotherapy is expanding as a novel treatment option for HCC. Emerging data from cohort 4 of the CA209–040 study, which investigated the safety and preliminary efficacy of nivolumab/ipilimumab co-administration in advanced HCC, suggest that the combination can be delivered safely with an acceptable proportion of reversible grade 3–4 toxicities (27.1%) and a low discontinuation rate (2%) in patients with HCC. Here, we describe the design and rationale of PRIME-HCC, a two-part, multi-centre, phase Ib study to assess safety and bioactivity of the nivolumab/ipilimumab combination prior to LR in early-stage HCC.

**Methods:**

The study involves an initial safety run-in phase (Part 1) to allow for preliminary safety characterisation within the first 6 patients enrolled and a subsequent expansion (Part 2). Ipilimumab will be administered once only on Day 1. Nivolumab will be administered on Day 1 and Day 22 (± 3 days) for a total of two 21-day cycles (i.e. 6 weeks of treatment). The primary objective of the study is to determine the safety and tolerability of the nivolumab/ipilimumab combination prior to LR. The secondary objective is to preliminarily characterize the efficacy of the combination prior to LR, including objective response rate (ORR) and pathologic response rates. Additional exploratory objectives include preliminary evidence of long-term disease control and to identify predictive correlates of response to the nivolumab/ipilimumab combination in HCC.

**Discussion:**

The results of this study will help define the positioning of neoadjuvant nivolumab/ipilimumab combination in the perioperative management of HCC, with potential to improve survival outcomes in this patient population.

**Trial registration:**

EudraCT Number: 2018–000987-27 Clinical trial registry & ID: ClinicalTrials.gov: NCT03682276.

**Supplementary Information:**

The online version contains supplementary material available at 10.1186/s12885-021-08033-x.

## Background

Hepatocellular carcinoma (HCC) is the sixth commonest and third most lethal solid malignancy on a global scale [1]. In spite of the major diagnostic advancements, only one out of three of the newly diagnosed patients are eligible for radical treatments upfront [2]. In patients within the Barcelona Clinic Liver Cancer (BCLC) stage 0 or A criteria, surgical resection can be carried out safely with curative intent [3]. However, this is only feasible in < 30% of the patients, mainly due to the extent of radiological spread of the disease or poor synthetic function due to underlying cirrhosis.

### The evolving landscape of systemic therapy for HCC

Anti-tumour immunotherapy with monoclonal antibodies blocking the programmed death-1/programmed death-ligand 1 (PD-1/PD-L1) and cytotoxic T-lymphocyte-associated protein 4 (CTLA-4) pathways are gaining momentum, where immune checkpoint inhibitors directed against these targets either alone or in combination have shown efficacy in HCC. Pembrolizumab has shown initial evidence of disease-modulating activity in a little less than 20% of patients enrolled in Keynote-224 [4], but failed to improve OS in Keynote-240, a placebo-controlled phase III trial which investigated pembrolizumab in treatment-experienced patients with advanced HCC [5]. CheckMate-040 (CA209–040) is the reference, open label, multi-cohort, phase I/II study which evaluated safety and efficacy of nivolumab in advanced HCC patients [6]. Despite evidence of anti-tumour activity, the subsequent phase III study CheckMate-459 failed to confirm thedif significant superiority of first-line nivolumab over sorafenib with respect of OS in the advanced disease (HR = 0.85, 95% CI: 0.72–1.02; *p* = 0.0752) [7].

Whilst not yet supported by evidence of OS improvement, dual CTLA-4/PD-1 blockade might also be beneficial, given the different and alternative role of the two biologic axes within the cancer immunity cycle, as CTLA-4 is a key driver in downregulation of tumour-antigen presenting cells and in T-regs enhancement, whereas PD-1/PD-L1 mainly impairs the efficiency of the CD8 + CTL response. Reliable evidence of synergistic effects from CTLA-4/PD-1 co-inhibition has been observed in clinical trials amongst other tumour types, in melanoma [8], renal cell carcinoma [9] and NSCLC [10].

In HCC, safety and preliminary efficacy of 3 alternative schedules of nivolumab and ipilimumab have been investigated within the cohort 4 of Checkmate-040. Pre-treated patients with Child-Pugh class A advanced disease have been randomised to receive either nivolumab 3 mg/kg + ipilimumab 1 mg/kg or nivolumab 1 mg/kg + ipilimumab 3 mg/kg or every 3 weeks for four doses followed by maintenance nivolumab monotherapy (240 mg flat dose biweekly) until disease progression or unacceptable toxicity. The further arm evaluated nivolumab 3 mg/kg + ipilimumab 1 mg/kg every 6 weeks until unacceptable toxicity or disease progression. The incidence of treatment related adverse events (TRAEs) for the whole cohort was 37% with a 5% discontinuation rate. The efficacy analysis showed the nivolumab/ipilimumab combination to achieve an ORR of 31%, which was higher than the ORR (14%) reported with single agent nivolumab in the same study, and led to US Food and Drug Administration approval of the nivolumab/ipilimumab combination in treatment-experienced advanced HCC patients [11].

### Neoadjuvant immunotherapy

The rapidly changing landscape of systemic therapy in HCC, where combination immunotherapy has led to reproducible evidence of disease-modulation, has prompted clinicians to consider the expansion of immunotherapy to the earlier stages of the disease. There is an acute need for highly active systemic anti-cancer treatments as a pre-operative strategy to attempt disease down-staging and expand the proportion of patients who might benefit from curative surgery. Furthermore, despite optimal local control of the disease, the majority of patients recur within 2 years of surgery as a likely result of proliferation of microscopic neoplastic foci within the residual organ, and the 5-year survival rate for early-stage HCC ranges between 17 to 53%, with a recurrence rat that can reach 70% also in the curative resection setting [2, 12]. The negative impact on survival secondary to both early and late recurrence of HCC suggests the need for an optimal integration of systemic anti-cancer therapies to control the risk of relapse and increase the chances of cure in patients with early stage HCC.

Currently, there is no standard of care for adjuvant/neoadjuvant treatment and although a number of approaches have been attempted including trans-arterial chemoembolization (TACE), Yttrium-90 radioembolization, and neoadjuvant sorafenib in HCC patients on waiting list for liver transplantation [13–15]. Failure to control micro-metastatic disease is a major determinant influencing early relapse and mortality following curative resection and randomized controlled trials have shown sorafenib, to be ineffective in improving outcomes in the adjuvant setting [16].

Here, we describe the design and the rationale of PRIME-HCC, a two-part multi-centre phase Ib study to assess safety and bioactivity of the nivolumab and ipilimumab combination before LR in patients with HCC.

## Methods/design

**Fig. 1:**
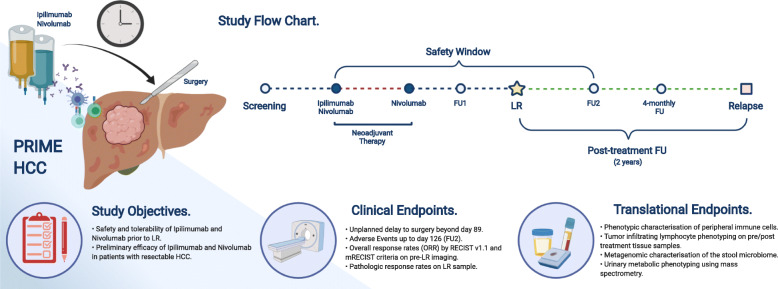
PRIME-HCC study objectives and timeline (original figure, no copyright permission required; Created with BioRender.com)

PRIME HCC is a two-part, phase Ib study of ipilimumab and nivolumab combination prior to LR in adult patients (aged ≥18 years) with HCC. Ipilimumab will be administered once only on Day 1. Nivolumab will be administered on Day 1 and Day 22 (± 3 days) for a total of two 21-day cycles (i.e. 6 weeks of treatment). Part 1 of the study will initially consist of a safety run-in phase of 6 participants. If two or fewer surgical delays are observed, the study will continue to Part 2, which will consist of an expansion phase including up to 26 participants. All participants entered into the study will receive nivolumab and ipilimumab for a fixed duration of 6 weeks of treatment. The study flow-chart is summarized in Fig. 1**.**

Ipilimumab is a fully human monoclonal immunoglobulin (Ig) G1k specific for human CTLA-4 manufactured by Bristol Myers Squibb (BMS). Nivolumab is a human monoclonal antibody (HuMAb; immunoglobulin G4 [IgG4]-S228P) manufactured by BMS that inhibits the PD-1 receptor on cell surface. The clinical safety profile of nivolumab and ipilimumab as a combination therapy has emerged from a number of clinical studies in a number of tumour types [17–22].

In PRIME-HCC the dosing schedule adopted is 1 mg/kg dose Q6W for ipilimumab in combination with nivolumab 3 mg/kg Q3W for a total of 2 cycles. The rationale for dose selection stems from the clinical experience in the use of the combination across a number of indications. In the specific setting of HCC, whilst there is currently no clinical data available to illustrate the safety and tolerability of the planned regimen administered sequentially with LR, similar dosing schedules including a six-weekly dosing of ipilimumab at 1 mg/kg and three-weekly dosing of nivolumab at 3 mg/kg have been explored in CA209–040 cohort 4. Extrapolating from CA209–040 safety data, ipilimumab dose intensity appears to be the strongest determinant of toxicity, in particular with regards to immune-related liver TRAEs: study treatment in Arms B and C, where ipilimumab dose intensitiy was lower, was shown to be better tolerable compared to more intense ipilimumab dosing schedules (3 mg/kg in Arm A). For this reason, PRIME-HCC will utilise the lowest effective dose intensity schedule (1 mg/kg Q6W) and combine it with nivolumab at the clinically approved dose of 3 mg/kg Q3W. The choice of this combination will maintain individual exposures that are optimally consistent with those associated with maximal efficacy response in other indications and that are tolerated and safe, especially considering liver-related TRAEs in a patient population where cirrhosis and concomitant chronic viral infection is highly prevalent.

### Eligibility criteria

Detailed eligibility criteria are provided in Table 1**.** In brief, PRIME-HCC will recruit patients amenable to resection and ineligible for liver transplantation, with a confirmed diagnosis of HCC by biopsy or by non-invasive diagnostic criteria of the American Association for the Study of the Liver (AASLD).

**Table 1:** Eligibility criteria of PRIME-HCC trial

Key inclusion criteria	Key exclusion criteria
1. Written informed consent for the trial 2. Aged ≥18 years 3. Confirmed diagnosis of HCC 4. Willing to provide tissue from an excisional biopsy of a tumour lesion 5. Have measurable disease by CT-scan or MRI defined by RECIST 1.1 criteria 6. Ineligible for liver transplantation 7. Medically fit to undergo surgery as determined by the treating medical and surgical oncology team. 8. ECOG-PS 0 or 1 9. Adequate hematologic function, defined as WBC ≥ 2000/μl, ANC ≥ 1500/μl, platelet count ≥50,000/μl and hemoglobin ≥8.5 g/dl without transfusion or Erythropoietin dependency. 10. Adequate renal function, defined as creatinine ≤1.5× ULN or measured or calculated creatinine clearance ≥40 ml/min for those with creatinine levels > 1.5× ULN 11. Adequate hepatic function, defined as total bilirubin ≤1.5× ULN, and ALT/AST levels ≤5× ULN, albumin ≥2.8 g/dL 12. Adequate coagulation function, defined as INR ≤ 1.5× ULN unless the patient is receiving anticoagulant therapy as long as PT or aPTT is within the therapeutic range 10. Overall Child-Pugh class A 11. Female patient of childbearing potential should have a negative serum pregnancy test within 24 h of her first dose of IMP 12. Women of childbearing potential must be willing to use a highly effective method of contraception 13. Sexually active males must agree to use an adequate method of contraception	1. Extrahepatic metastasis 2. Prior systemic anticancer treatment for HCC, including an anti-PD-1, anti-PD-L1 or anti-CTLA-4 antibody 3. Prior orthotopic liver transplantation 4. Any major surgery within the 3 weeks prior to enrolment 5. Hepatic encephalopathy 6. Ascites that is refractory to diuretic therapy 7. Is currently receiving anti-cancer therapy (chemotherapy, radiation therapy, immunotherapy or biologic therapy) or has participated or is participating in a study with Nivolumab or Ipilimumab or used an investigational device within 4 weeks of the first dose of IMP 8. Diagnosis of immunodeficiency or is receiving systemic steroid therapy or any other form of immunosuppressive therapy 9. Known history of active Bacillus Tuberculosis 10. History of known hypersensitivity to any monoclonal antibody or any of their excipients 11. Known additional malignancy that is progressing or requires active treatment ^a^ 12. Active autoimmune disease that has required systemic treatment in the past 2 years ^b^ 13. Known history of, or any evidence of active, non-infectious pneumonitis 14. Active infection requiring systemic therapy ^c^ 15. History or current evidence of any condition, therapy, or laboratory abnormality that might confound the results of the trial 16. Known psychiatric or substance abuse disorders that would interfere with cooperation with the requirements of the trial 17. Pregnant or breastfeeding 18. Known history of Human Immunodeficiency Virus (HIV; HIV 1/2 antibodies) 19. Received a live vaccine within 30 days of first dose of IMP

*CT* Computed tomography; *MRI* Magnetic resonance imaging; *ECOG-PS* Eastern cooperative oncology group-performance status; *WBC* White blood cells; *ANC* Absolute neutrophil count; *ULN* Upper limit of normal; *ALT/AST* Alanine/aspartate aminotransferase; *IMP* Investigational medicinal product; *PTT/PT* Partial thromboplastin time/prombin time; ^a^ Exceptions include basal cell carcinoma of the skin or squamous cell carcinoma of the skin that has undergone potentially curative therapy, or in situ cervical cancer; ^b^replacement therapy (eg. thyroxine, insulin, or physiologic corticosteroid replacement therapy for adrenal or pituitary insufficiency, etc.) is not considered a form of systemic treatment; ^c^ with the exceptions relating to Hepatitis B and C virus

Participants will require a full hepatitis serology screen prior to enrolment into the study, this includes Hepatitis B and Hepatitis C Virus serology. In patients with positive serology for either virus, baseline HBV DNA and HCV quantitative RNA levels will be requested. Participants who are confirmed to have chronic and active hepatitis B and/or C (i.e. with detectable HBV DNA or HCV RNA at baseline) will have their viral load (HCV RNA and/or HBV DNA as appropriate) monitored at each cycle and at the end of treatment follow-up visit.

### Planned sample size and study period

We designed the study as a safety-oriented trial and considered enrolling a cohort of up to 32 participants to:

1. Comprehensively characterise the safety profile of nivolumab and ipilimumab co-administration in patients with early-stage HCC;
2. Preliminarily document the efficacy of treatment to inform subsequent efficacy-testing in a future adequately powered trial;
3. Obtain adequate information regarding the disease-modulating effects from treatment reflected by the proposed exploratory endpoints.

With the primary endpoint being safety, no power calculation for hypothesis testing is required to formally power the study: the upper 95% confidence interval for toxicity events will inform the decision to proceed to a future, adequately powered, phase II trial. Part 1 of the study will initially require a total of 6 patients. If ≤2 surgical. Delays are observed (primary safety endpoint), the study will continue to Part 2 following Independent Safety Data Monitoring Committee review of preliminary safety data. A second interim analysis will be performed when 100% of the recruited patients have completed study follow-up visit 2 (FU2) to evaluate general safety and tolerability of the combination.

### Study procedures

Patients will undergo baseline tumour imaging including computed tomography (CT) scan of the chest, abdomen and pelvis, and by contrast enhanced magnetic resonance imaging (MRI) scan of the liver at screening. At post-treatment time-points prior to surgery (on Day 43), after surgery (on Day 127) and 4-monthly thereafter, tumour imaging will be repeated using contrast enhanced MRI. A triple-phase CT of the liver is an acceptable alternative for intrahepatic staging in patients with contraindications to MRI. Baseline CT/MRI scans do not need to be repeated if obtained within 35 days of first dose. The same method used for assessment at baseline must then be used at all subsequent time points. RECIST v1.1 criteria will be used to determine patient response to treatment, progression-free survival (PFS) and ORR. A baseline core tumor biopsy will be collected from participants at screening and patients will have a parcel of leftover tissue from their LR specimen stored for correlative studies. Treatment will consist of a maximum of 2 cycles of nivolumab 3 mg/kg and 1 cycle of ipilimumab 1 mg/kg administered intravenously Q3W. Patients will be reviewed following completion of IMP treatment (Follow-up visit 1; FU1) on Day 43 +/− 3, prior to surgery. LR will be performed as per standard of care. The safety FU2 will be conducted 126 days (± 7 days) after the first dose of the investigational medicinal product (IMP). All AEs that occur prior to the visit will be recorded. Participants with on-going AEs at the visit will be followed up by principal investigator (PI) or delegate until resolution or stabilisation of the event. Following FU2, participants will be assessed every 4 months (± 7 days) thereafter to collect information regarding disease status and survival. Long-term follow-up will continue, for each patient, for a total of 2 years. The Standard Protocol Items: Recommendations for Interventional Trials (SPIRIT) schedule of enrolment, interventions, and assessments is provided in Supplementary Table 1**.**

### Outcome measures and endpoints

Primary study endpoints include rate of patients experiencing a surgery delay to Day 89 or later and determination of safety and tolerability of the nivolumab/ipilimumab combination based on National Cancer Institute Common Terminology Criteria for Adverse Events (NCI CTCAE) v5.0 criteria from nivolumab and ipilimumab initiation to 126 days later.

Secondary endpoints include ORR on pre-resection imaging at day 42 according to the RECIST v1.1 criteria and pathologic response rate on evaluation of the resected specimen. Exploratory endpoints are PFS rate and OS at day 126 and every 4 months thereafter and identity of biomarkers of response to nivolumab and ipilimumab using high-throughput technologies.

### Adverse events management and dose modifications

Adverse events (both non-serious and serious) of unknown aetiology, associated with IMP exposure and likely to have an immune-mediated underlying mechanism may be considered as irAEs. These AEs may occur from soon after the first dose to months after the last dose. If an irAE is suspected, efforts will be put in place to exclude any other etiologic causes prior to labelling an AE as an irAEs. Summaries of recommendations to managing irAEs and of the physician’s guidelines for IMP’s temporary interruption and permanent discontinuation in case of toxicities are provided in Table 2 and Table 3**,** respectively**.** Treatment discontinuation for AST, ALT deterioration in participants who present with Grade 2 AST or ALT at Cycle 1 Day 1 will be triggered only if ALT or AST increase by more than or equal to 50% compared to the measurement taken at initiation of systemic treatment (Cycle 1 Day 1) and lasts for at least 1 week. In addition to the guidelines, dosing should be permanently discontinued in any participant who does not receive the Cycle 2 dose within the allowed window (Day 18 to Day 24) due to being unfit for dosing for any reason.

**Table 2:** Summary of recommendations to managing immune-related adverse events. IrAEs: immune-related adverse events; IMP: investigational medicinal product

irAE	Supportive Care Intervention
Grade 1	Provide symptomatic treatment
Grade 2	Consider systemic corticosteroids in addition to appropriate symptomatic treatment. Steroid taper should be considered once symptoms improve to Grade 1 or less and tapered over at least 4 weeks.
Grade 3 and Grade 4	Systemic corticosteroids are indicated in addition to appropriate symptomatic treatment. May utilize 1 to 2 mg/kg prednisone or equivalent per day. Steroid taper should be considered once symptoms improve to Grade 1 or less and tapered over at least 4 weeks. Consider referral to organ-specific specialist (i.e. gastroenterologist, hepatologist, respiratory physician, endocrinologist) for any grade 3–4 irAEs, in order to evaluate substitution treatments and immune-modulating agents, such as anti-TNFα of other immune suppressants.

**Table 3:** Summary of the physicians’ guidelines for IMP’s temporary interruption and permanent discontinuation in case of immune-related adverse events. PI: principal Investigator

Toxicity	Hold treatment for grade	Timing for restarting treatment
Diarrhoea/ Colitis	2–3	Toxicity resolves to Grade 0–1
	4	Permanently discontinue
AST, ALT, or Increased Bilirubin	2	Toxicity resolves to Grade 0–1
	3–4	Permanently discontinue (see exception below)^a^
Type 1 diabetes mellitus (if new onset) or Hyperglycaemia	T1DM or 3–4	Hold the IMP for new onset Type 1 diabetes mellitus or Grade 3–4 hyperglycemia associated with evidence of beta cell failure
Hypophysitis	2–4	Toxicity resolves to Grade 0–1. Therapy with IMP can be continued while endocrine replacement therapy is instituted
Hyperthyroidism	3	Toxicity resolves to Grade 0–1
	4	Permanently discontinue
Hypothyroidism	N/A	Therapy with IMP can be continued while thyroid replacement therapy is instituted
Infusion Reaction	2^b^	Toxicity resolves to Grade 0–1
	3–4	Permanently discontinue
Pneumonitis	2	Toxicity resolves to Grade 0–1
	3–4	Permanently discontinue
Renal Failure or Nephritis	2	Toxicity resolves to Grade 0–1
	3–4	Permanently discontinue
All Other Drug-Related Toxicity^c^	3 or Severe	Toxicity resolves to Grade 0–1
	4	Permanently discontinue

Note: Permanently discontinue for any severe or Grade 3 drug-related AE that recurs or any life-threatening event

^a^ For participants who begin treatment with Grade 2 AST or ALT at Cycle 1 Day 1 of treatment, if AST or ALT increases by greater than or equal to 50% relative to baseline and lasts for at least 1 week then participants should be discontinued

^b^ If symptoms resolve within one hour of stopping IMP infusion, the infusion may be restarted at 50% of the original infusion rate (e.g., from 100 mL/hr. to 50 mL/hr). Otherwise dosing will be held until symptoms resolve and the participant should be pre-medicated for the next scheduled dose

^c^ Participants with intolerable or persistent Grade 2 drug-related AE may hold study medication at PI/delegate discretion

### Statistical analysis

Statistical analyses will include an intent to treat (ITT) analysis including all participants enrolled and a per protocol analysis using all participants who complete the study without major protocol violations. Descriptive analyses will be performed by cross-tabulating relevant predictors (e.g. demographic variables) and endpoints. Appropriate statistical tests will be carried out, such as Fisher’s exact test for categorical variables and Wilcoxon tests for continuous variables. Univariable regression analyses will also be performed to estimate regression coefficients and their 95% confidence intervals. Data analysis will occur when the study is complete. Interim analyses of safety data will be conducted at the end of FU1 and at the end of FU2. The comprehensive statistical analysis plan will be finalised prior to the final analysis.

All participants who receive at least one dose of IMP will be included in the safety analysis set. All participants who receive at least one dose of the IMP and undergo disease re-evaluation as per protocol will be included in the efficacy analysis. Due to the exploratory nature of the study with regards to efficacy endpoints, pathologic response rates and RECIST 1.1 response rates will be presented descriptively. The exploratory endpoint of PFS rate will be shown with Kaplan-Meier plots using the full timespan from the start of IMP administration until the date of progression or death from any cause. Proportions of participants who do not progress and who are alive at Day 127 (FU2) and 4-monthly time-points thereafter will also be estimated. For the exploratory endpoints, appropriate descriptive analysis and univariable regression analysis will be conducted.

### Translational endpoints

Alongside primary clinical endpoints, PRIME-HCC will generate a biorepository of peripheral blood, tissue, stool and urine samples collected at different study timepoints (Supplementary Table 2). Tissue samples pre- and post-immunotherapy will undergo multi-colour immune profiling to evaluate the expression of PD ligands and a panel of other immune checkpoints evaluated in co-expression with CD4, CD8, CD20, CD56, CD68 and FoxP3 to verify their immunologic role in relationship to the peri-tumoural infiltrate. Following macro-dissection and total RNA extraction, targeted transcriptomic profiling using the Nanostring PanCancer Immune panel will be used to evaluate enrichment of 770 genes pertaining to 24 immune cell types in pre-and post-treatment tissue samples. Peripheral blood mononuclear cells will be profiled using flow cytometry to differentiate relative abundance T-cytotoxic (CD3^+^/CD8^+^), T-helper (CD3^+^/CD4^+^/CD25^−^) and T-reg cells (CD3^+^/CD4^+^/CD25^high^) and downstream transcriptome analysis will decipher the relative functional contribution of individual T-cell subsets in influencing response to ICPI. Lastly, we will perform plasma and urinary metabolic phenotyping and stool metagenomic analysis to evaluate the contribution of the gut microbiota and associated metabolites in influencing response to neoadjuvant ICI therapy.

## Discussion

PD-1/PD-L1 inhibitors and CTLA-4 inhibitors, either alone or in combination represent a promising peri-operative therapeutic option. A single dose of pre-operative pembrolizumab, can induce near complete pathologic responses in 19% of stage III/IV resectable melanoma patients [23]. Preliminary efficacy reports suggest even higher rates of complete pathologic response ranging from 7 to 45% from neoadjuvant nivolumab/ipilimumab therapy [24]. In NSCLC, neoadjuvant checkpoint blockade leads to a higher proportion of major pathologic responses compared to chemotherapy [25] and the phase II NEOSTAR trial, has shown that treatment with nivolumab/ipilimumab leads to higher major pathological responses (33%) compared to nivolumab (17%) [26]. Similar positive results have been seen in non-metastatic muscle-invasive bladder cancer, where two studies have reported complete response rates of 40 and 29% after up to 3 doses of anti-PD-1/PD-L1 monotherapy [27, 28]. A recent phase Ib study of sequential nivolumab/ipilimumab followed by surgery reported pathological complete responses in 45% of patients, although 54% of the patients experienced grade 3–4 immune-related adverse events (irAEs) [29]. Importantly, alongside the efficacy data, no unexpected surgical delays/complications were reported, and the safety profile appeared improved over chemotherapy also in the neoadjuvant setting [23, 25, 26, 29].

The study of immune-checkpoint inhibitors in the preoperative setting of HCC is an ideal scenario to enable comprehensive evaluation of predictive correlates of response to treatment. Therapeutic expansion of immunotherapy to early stage patients, however, is accompanied by a number of clinical concerns that require to be addressed prospectively. Firstly, from a safety point of view, concurrent liver failure stemming from underlying cirrhosis and active hepatotropic viral infection makes the interaction between immune checkpoint inhibition and surgical resection a clinical scenario deserving comprehensive safety evaluation. Secondly, there is an inherent lack of efficacy data to confirm the sensitivity of early stage, treatment-naïve HCC patients to immune checkpoint inhibition. This is a highly relevant point in generalizing the efficacy of anti-PD-1/PD-L1 and anti-CTLA-4 agents across all the stages of HCC, especially given that the majority of patients that have been previously recruited to clinical trials of immune checkpoint inhibitors may have had loco-regional therapies (radiofrequency ablation or TACE). These treatments are widely renowned inducers of immunogenic cell death that might have exerted a priming effect on the immune system by facilitating a broader access to otherwise inaccessible neo-epitopes. Confirmation of the bioactivity of nivolumab in combination with ipilimumab in early stage HCC, where the biology of the disease may significantly differ from that of relapsed or advanced disease, is therefore an important research aim to allow optimal allocation of checkpoint blockade in the treatment paradigm of patients with HCC.

Despite optimal surgical control of the disease, patients with HCC are at high risk of recurrence after LR. Currently, there is no standard of care for neoadjuvant therapy, although a number of approaches have been attempted. The study of immune-checkpoint inhibitors in the preoperative setting of HCC is an ideal scenario to enable comprehensive evaluation of predictive correlates of response to anti-PD-1 and anti-CTLA-4 checkpoint inhibitors. The results of this study will help to define the role of neoadjuvant nivolumab/ipilimumab combination in the management of HCC, with potential to improve survival outcomes in this patient population.

## Supplementary Information

**Additional file 1.**

**Additional file 2.**

## Data Availability

Not applicable.

## Abbreviations

HCC: Hepatocellular carcinoma
LR: Liver resection
ORR: Objective response rate
BCLC: Barcelona clinic liver cancer
TACE: Trans-arterial chemoembolization
PD-1: Programmed death-1
PD-L1: Programmed death-ligand 1
CTLA-4: Cytotoxic T-lymphocyte-associated protein 4
PFS: Progression free survival
OS: Overall survival
TRAEs: Treatment related adverse events
NSCLC: Non-small cell lung cancer
US: United States
EU: European union
AASLD: American association for the study of the liver
IDSMC: Independent safety data monitoring committee
FU1: Follow-up visit 1
FU2: Follow-up visit 2
CT: Computed tomography
MRI: Magnetic resonance imaging
SPIRIT: Standard protocol items: recommendations for interventional trials
IMP: Investigational medicinal product
NCI CTCAE: National cancer institute common terminology criteria for adverse events
IrAEs: Immune-related adverse events
ITT: Intent to treat

## References

[1] Parkin DM, Bray F, Ferlay J, Pisani P (2005). Global cancer statistics, 2002. CA Cancer J Clin.

[2] Ercolani G, Grazi GL, Ravaioli M, del Gaudio M, Gardini A, Cescon M, Varotti G, Cetta F, Cavallari A (2003). Liver resection for hepatocellular carcinoma on cirrhosis: univariate and multivariate analysis of risk factors for intrahepatic recurrence. Ann Surg.

[3] Bruix J, Sherman M (2005). Practice guidelines committee, American Association for the Study of Liver Diseases. Management of hepatocellular carcinoma. Hepatology.

[4] Zhu AX, Finn RS, Edeline J, Cattan S, Ogasawara S, Palmer D, Verslype C, Zagonel V, Fartoux L, Vogel A, Sarker D, Verset G, Chan SL, Knox J, Daniele B, Webber AL, Ebbinghaus SW, Ma J, Siegel AB, Cheng AL, Kudo M, Alistar A, Asselah J, Blanc JF, Borbath I, Cannon T, Chung K, Cohn A, Cosgrove DP, Damjanov N, Gupta M, Karino Y, Karwal M, Kaubisch A, Kelley R, van Laethem JL, Larson T, Lee J, Li D, Manhas A, Manji GA, Numata K, Parsons B, Paulson AS, Pinto C, Ramirez R, Ratnam S, Rizell M, Rosmorduc O, Sada Y, Sasaki Y, Stal PI, Strasser S, Trojan J, Vaccaro G, van Vlierberghe H, Weiss A, Weiss KH, Yamashita T (2018). Pembrolizumab in patients with advanced hepatocellular carcinoma previously treated with sorafenib (KEYNOTE-224): a non-randomised, open-label phase 2 trial. Lancet Oncol.

[5] Finn RS, Ryoo BY, Merle P, Kudo M, Bouattour M, Lim HY, Breder V, Edeline J, Chao Y, Ogasawara S, Yau T, Garrido M, Chan SL, Knox J, Daniele B, Ebbinghaus SW, Chen E, Siegel AB, Zhu AX, Cheng AL, on behalf of the KEYNOTE-240 investigators (2020). Pembrolizumab as second-line therapy in patients with advanced hepatocellular carcinoma in KEYNOTE-240: a randomized, double-blind, phase III trial. J Clin Oncol.

[6] El-Khoueiry AB, Sangro B, Yau T, Crocenzi TS, Kudo M, Hsu C (2017). Nivolumab in patients with advanced hepatocellular carcinoma (CheckMate 040): an open-label, non-comparative, phase 1/2 dose escalation and expansion trial. Lancet.

[7] Yau T, Park JW, Finn RS, Cheng A-L, Mathurin P, Edeline J (2019). LBA38_PRCheckMate 459: a randomized, multi-center phase III study of nivolumab (NIVO) vs sorafenib (SOR) as first-line (1L) treatment in patients (pts) with advanced hepatocellular carcinoma (aHCC). Ann Oncol.

[8] Larkin J, Chiarion-Sileni V, Gonzalez R, Grob JJ, Rutkowski P, Lao CD, Cowey CL, Schadendorf D, Wagstaff J, Dummer R, Ferrucci PF, Smylie M, Hogg D, Hill A, Márquez-Rodas I, Haanen J, Guidoboni M, Maio M, Schöffski P, Carlino MS, Lebbé C, McArthur G, Ascierto PA, Daniels GA, Long GV, Bastholt L, Rizzo JI, Balogh A, Moshyk A, Hodi FS, Wolchok JD (2019). Five-year survival with combined Nivolumab and Ipilimumab in advanced melanoma. N Engl J Med.

[9] Motzer RJ, Rini BI, McDermott DF, Arén Frontera O, Hammers HJ, Carducci MA, Salman P, Escudier B, Beuselinck B, Amin A, Porta C, George S, Neiman V, Bracarda S, Tykodi SS, Barthélémy P, Leibowitz-Amit R, Plimack ER, Oosting SF, Redman B, Melichar B, Powles T, Nathan P, Oudard S, Pook D, Choueiri TK, Donskov F, Grimm MO, Gurney H, Heng DYC, Kollmannsberger CK, Harrison MR, Tomita Y, Duran I, Grünwald V, McHenry M, Mekan S, Tannir NM, CheckMate 214 investigators (2019). Nivolumab plus ipilimumab versus sunitinib in first-line treatment for advanced renal cell carcinoma: extended follow-up of efficacy and safety results from a randomised, controlled, phase 3 trial [published correction appears in lancet Oncol. 2019 Aug 21;:] [published correction appears in lancet Oncol. 2020 Jun;21(6):e304]. Lancet Oncol.

[10] Hellmann MD, Paz-Ares L, Bernabe Caro R, Zurawski B, Kim SW, Carcereny Costa E, Park K, Alexandru A, Lupinacci L, de la Mora Jimenez E, Sakai H, Albert I, Vergnenegre A, Peters S, Syrigos K, Barlesi F, Reck M, Borghaei H, Brahmer JR, O’Byrne KJ, Geese WJ, Bhagavatheeswaran P, Rabindran SK, Kasinathan RS, Nathan FE, Ramalingam SS (2019). Nivolumab plus Ipilimumab in advanced non-small-cell lung Cancer. N Engl J Med.

[11] Yau T, Kang Y-K, Kim T-Y, El-Khoueiry AB, Santoro A, Sangro B (2019). Nivolumab (NIVO)+ ipilimumab (IPI) combination therapy in patients (pts) with advanced hepatocellular carcinoma (aHCC): results from CheckMate 040. J Clin Oncol.

[12] Chen XP, Qiu FZ, Wu ZD, Zhang ZW, Huang ZY, Chen YF (2006). Long-term outcome of resection of large hepatocellular carcinoma. Br J Surg.

[13] Kang WH, Hwang S, Song GW, Lee YJ, Kim KH, Ahn CS, Moon DB, Jung DH, Park GC, Lee SG (2017). Prognostic effect of transarterial chemoembolization-induced complete pathological response in patients undergoing liver resection and transplantation for hepatocellular carcinoma. Liver Transpl.

[14] Toso C, Mentha G, Kneteman NM, Majno P (2010). The place of downstaging for hepatocellular carcinoma. J Hepatol.

[15] Hoffmann K, Ganten T, Gotthardtp D, Radeleff B, Settmacher U, Kollmar O, Nadalin S, Karapanagiotou-Schenkel I, von Kalle C, Jäger D, Büchler MW, Schemmer P (2015). Impact of neo-adjuvant Sorafenib treatment on liver transplantation in HCC patients - a prospective, randomized, double-blind, phase III trial. BMC Cancer.

[16] Bruix J, Takayama T, Mazzaferro V, Chau GY, Yang J, Kudo M, Cai J, Poon RT, Han KH, Tak WY, Lee HC, Song T, Roayaie S, Bolondi L, Lee KS, Makuuchi M, Souza F, Berre MAL, Meinhardt G, Llovet JM (2015). Adjuvant sorafenib for hepatocellular carcinoma after resection or ablation (STORM): a phase 3, randomised, double-blind, placebo-controlled trial. Lancet Oncol.

[17] Antonia SJ, López-Martin JA, Bendell J, Ott PA, Taylor M, Eder JP, Jäger D, Pietanza MC, le DT, de Braud F, Morse MA, Ascierto PA, Horn L, Amin A, Pillai RN, Evans J, Chau I, Bono P, Atmaca A, Sharma P, Harbison CT, Lin CS, Christensen O, Calvo E (2016). Nivolumab alone and nivolumab plus ipilimumab in recurrent small-cell lung cancer (CheckMate 032): a multicentre, open-label, phase 1/2 trial. Lancet Oncol.

[18] Hellmann MD, Rizvi NA, Goldman JW, Gettinger SN, Borghaei H, Brahmer JR, Ready NE, Gerber DE, Chow LQ, Juergens RA, Shepherd FA, Laurie SA, Geese WJ, Agrawal S, Young TC, Li X, Antonia SJ (2017). Nivolumab plus ipilimumab as first-line treatment for advanced non-small-cell lung cancer (CheckMate 012): results of an open-label, phase 1, multicohort study. Lancet Oncol.

[19] Hammers HJ, Plimack ER, Infante JR, Rini BI, McDermott DF, Lewis LD, Voss MH, Sharma P, Pal SK, Razak ARA, Kollmannsberger C, Heng DYC, Spratlin J, McHenry MB, Amin A (2017). Safety and efficacy of Nivolumab in combination with Ipilimumab in metastatic renal cell carcinoma: the CheckMate 016 study. J Clin Oncol.

[20] Janjigian YY, Bendell J, Calvo E, Kim JW, Ascierto PA, Sharma P, Ott PA, Peltola K, Jaeger D, Evans J, de Braud F, Chau I, Harbison CT, Dorange C, Tschaika M, le DT (2018). CheckMate-032 study: efficacy and safety of nivolumab and nivolumab plus ipilimumab in patients with metastatic esophagogastric cancer [published correction appears in J Clin Oncol. 2019 Feb 10;37(5):443]. J Clin Oncol.

[21] Morse MA, Overman MJ, Hartman L, Khoukaz T, Brutcher E, Lenz HJ, Atasoy A, Shangguan T, Zhao H, el-Rayes B (2019). Safety of Nivolumab plus low-dose Ipilimumab in previously treated microsatellite instability-high/mismatch repair-deficient metastatic colorectal Cancer. Oncologist..

[22] Omuro A, Vlahovic G, Lim M, Sahebjam S, Baehring J, Cloughesy T, Voloschin A, Ramkissoon SH, Ligon KL, Latek R, Zwirtes R, Strauss L, Paliwal P, Harbison CT, Reardon DA, Sampson JH (2018). Nivolumab with or without ipilimumab in patients with recurrent glioblastoma: results from exploratory phase I cohorts of CheckMate 143. Neuro-Oncology.

[23] Huang AC, Orlowski RJ, Xu X, Mick R, George SM, Yan PK, Manne S, Kraya AA, Wubbenhorst B, Dorfman L, D’Andrea K, Wenz BM, Liu S, Chilukuri L, Kozlov A, Carberry M, Giles L, Kier MW, Quagliarello F, McGettigan S, Kreider K, Annamalai L, Zhao Q, Mogg R, Xu W, Blumenschein WM, Yearley JH, Linette GP, Amaravadi RK, Schuchter LM, Herati RS, Bengsch B, Nathanson KL, Farwell MD, Karakousis GC, Wherry EJ, Mitchell TC (2019). A single dose of neoadjuvant PD-1 blockade predicts clinical outcomes in resectable melanoma. Nat Med.

[24] Garutti M, Buriolla S, Bertoli E (2020). "To Anticipate": neoadjuvant therapy in melanoma with a focus on predictive biomarkers. Cancers (Basel).

[25] Jia XH, Xu H, Geng LY (2020). Efficacy and safety of neoadjuvant immunotherapy in resectable nonsmall cell lung cancer: a meta-analysis [published online ahead of print, 2020 Jul 10]. Lung Cancer.

[26] Cascone T, William WN, Weissferdt A, Lin HY, Leung CH, Carter BW, Fossella FV, Mott F, Papadimitrakopoulou V, Blumenschein GR, le X, Federico L, Parra Cuentas ER, Bernatchez C, Wistuba II, Vaporciyan AA, Gibbons DL, Swisher S, Heymach J, Sepesi B, NEOSTAR Study Group (2019). Neoadjuvant nivolumab (N) or nivolumab plus ipilimumab (NI) for resectable non-small cell lung cancer (NSCLC): clinical and correlative results from the NEOSTAR study. J Clin Oncol.

[27] Necchi A, Anichini A, Raggi D, Briganti A, Massa S, Lucianò R, Colecchia M, Giannatempo P, Mortarini R, Bianchi M, Farè E, Monopoli F, Colombo R, Gallina A, Salonia A, Messina A, Ali SM, Madison R, Ross JS, Chung JH, Salvioni R, Mariani L, Montorsi F (2018). Pembrolizumab as Neoadjuvant therapy before radical cystectomy in patients with muscle-invasive Urothelial bladder carcinoma (PURE-01): an open-label, single-arm. Phase II Study J Clin Oncol.

[28] Powles T, Rodriguez-Vida A, Duran I et al. A phase II study investigating the safety and efficacy of neoadjuvant atezolizumab in muscle invasive bladder cancer (ABACUS). J Clin Oncol 36(Suppl.): Abstract 4506.

[29] Van der Heijden MS, van Dijk N, Smit L (2019). Pre-operative ipilimumab and nivolumab in locoregionally advanced, stage III, urothelial cancer (NABUCCO). Ann Oncol.

