# Supplementary material for: PRIME-HCC: phase Ib study of neoadjuvant ipilimumab and nivolumab prior to liver resection for hepatocellular carcinoma
Source: BMC Cancer. 2021 Mar 23;21:301. doi: 10.1186/s12885-021-08033-x (PMC7988931; doi:10.1186/s12885-021-08033-x)
Supplement: Supplementary file 1 — Additional file 1. [file 12885_2021_8033_MOESM1_ESM.docx]

|  | **Screening** | **Treatment Cycles^1^** | | **LR** | | **Post-Treatment** | |
| --- | --- | --- | --- | --- | --- | --- | --- |
| Treatment Cycle Nos  Week Nos |  | 1  Wk 1 | 2  Wk 4 | FU1 | LR | FU2^13^ | Long-term Follow-Up Visits |
| Study Day Nos (window): | -28 to 0 | 1 | 22 (±3 d) | 43 (±3 d) | 44-85 (±3 d) | Day 127 (±7 d) | Q4M (±7 d) |
| Informed Consent | X |  |  |  |  |  |  |
| Demographics and Medical History | X |  |  |  |  |  |  |
| Tumour Imaging: CT Chest Abdomen and Pelvis ^2^ | X |  |  |  |  |  |  |
| Tumour Imaging: MRI Liver | X |  |  | X |  | X | X |
| Assessment of Child-Pugh Score | X | X | X | X |  | X |  |
| Full Physical Examination | X |  |  |  |  |  |  |
| Directed Physical Examination |  | (X) | (X) | (X) |  | (X) |  |
| Vital Signs, Weight & Height^11^ | X^11^ | X | X | X |  | X |  |
| ECOG Performance Status | X | X | X | X |  | X |  |
| 12–Lead ECG | X |  |  | X |  |  |  |
| Pregnancy Test – Serum beta-HCG^12^ | X |  |  |  |  |  |  |
| Hepatitis Serology^3^ | X |  |  |  |  |  |  |
| Viral Load (HBV DNA, HCV RNA)^4^ |  | (X) | (X) | (X) |  | (X) |  |
| PT/INR and aPTT | X | X | X | X |  | X |  |
| Alpha fetoprotein | X | X | X | X |  | X |  |
| Haematology^5^ | X | X | X | X |  | X |  |
| Biochemistry^6^ | X | X | X | X |  | X |  |
| Urinalysis^7^ | X | X | X | X |  | X |  |
| T3, T4 and TSH | X | X | X | X |  | X |  |
| Liver Resection |  |  |  |  | X |  |  |
| Nivolumab Administration |  | X | X |  |  |  |  |
| Ipilimumab Administration |  | X |  |  |  |  |  |
| Archival Tissue Collection | X |  |  |  |  |  |  |
| Biomarker Biopsy Sample^8^ | X |  |  |  |  |  |  |
| Biomarker Resection Specimen collection^9^ |  |  |  |  | X |  |  |
| Record Prior/Concomitant Medications | X | X | X | X |  | X |  |
| AE Assessment (NCI-CTCAE v5.0) | X | X | X | X |  | X |  |
| Post-study anticancer therapy status |  |  |  |  |  |  | X |
| Optional Biomarker Blood Sample^10^ | X | X |  | X |  |  |  |
| Optional Biomarker Circulating Tumour Cells (CTCs) Sample^10^ | X |  |  | X |  |  |  |
| Optional Biomarker Urine Sample^10^ | X | X |  | X |  |  |  |
| Optional Biomarker Stool Sample^10^ | X |  |  | X |  |  |  |
