# Supplementary material for: PRIME-HCC: phase Ib study of neoadjuvant ipilimumab and nivolumab prior to liver resection for hepatocellular carcinoma
Source: BMC Cancer. 2021 Mar 23;21:301. doi: 10.1186/s12885-021-08033-x (PMC7988931; doi:10.1186/s12885-021-08033-x)
Supplement: Supplementary file 2 — Additional file 2. [file 12885_2021_8033_MOESM2_ESM.docx]

|  | **Screening** | **Treatment Cycles** | | **Liver Resection** | | **Post Treatment** | |
| --- | --- | --- | --- | --- | --- | --- | --- |
| Treatment Cycle Nos  Week Nos | Screening | 1  Wk1 | 2  Wk4 | FU1 | LR | FU2 | Long-term follow-up visits |
| Scheduling Window  (Days): | -28 to 0 | 1 | 22  (±3 d) | 43 (±3d) | 44-85 (±3d) | Day 127 (±7d) | Q4M (±7d) |
| Archival Tissue Collection | X |  |  |  |  |  |  |
| Biomarker Fresh Tumour Biopsy Sample | X |  |  |  |  |  |  |
| Biomarker Blood Sample | X | X |  | X |  |  |  |
| Circulating Tumour Cells (CTCs) | X |  |  | X |  |  |  |
| Biomarker Urine Sample | X | X |  | X |  |  |  |
| Biomarker Stool Sample | X |  |  | X |  |  |  |
